# Change in woody cover at representative sites in the Kruger National Park, South Africa, based on historical imagery

**DOI:** 10.1186/s40064-016-3036-1

**Published:** 2016-08-24

**Authors:** C. Munyati, N. I. Sinthumule

**Affiliations:** 1 Department of Geography and Environmental Science, North-West University (Mafikeng Campus), Private Bag X2046, Mmabatho, 2735 South Africa; 2 Department of Ecology and Resource Management, University of Venda, Private Bag X2046, Thohoyandou, 0950 South Africa

**Keywords:** Savanna ecology, Woody vegetation, Remote sensing, Change detection, GIS

## Abstract

**Background:**

The coexistence of woody vegetation and grass is a key characteristic of savanna ecological balance. Gains in woody vegetation at the expense of grass can lead to changes in grazer and browser carrying capacities on the savannas. This study examined long-term change in woody cover at four study sites representative of the geology and rainfall in the Kruger National Park, South Africa. Scanned 1940/1942, 1968, and 1977 high spatial resolution (0.44–1.35 m) panchromatic aerial photographs were used, supplemented by 5 and 10 m resolution 1998 and 2012 panchromatic and red band grey scale digital SPOT images. The imagery datasets of the respective study sites were georeferenced to the UTM projection. Woody cover on the imagery was enhanced using texture analysis, and mapped by unsupervised classification of the texture images using the *K*-means clustering algorithm. Change in woody cover was mapped using Boolean addition Geographic Information System overlay analysis.

**Results:**

The results indicated 29 and 40 % reductions in woody cover for the southern granites and southern basalts sites, respectively. The northern granites and northern basalts sites, on the other hand, had gains in woody cover over the analysis period. The location context-specific factors of fire frequency and elephant density, and not rainfall fluctuations, explained most of the change in woody cover.

**Conclusions:**

The results point to the need for location context-specific management of fire and elephant concentrations. The changes in woody cover are likely to have effects on the grazer and browser carrying capacities of the savannas in the Kruger National Park.

## Background

Savannas are characterised by a coexistence of woody vegetation and grass (Higgins et al. 2007; Accatino et al. 2010), in inter-specific competition (Knoop and Walker 1985; Skarpe 1991; Bond and Midgeley 2000). They support a large diversity of ungulate species (du Toit and Cumming 1999), many of which are grazers. An increase in savanna woody vegetation at the expense of grass can, therefore, potentially result in reduced carrying capacity for grazers in favour of browsers, and vice versa. As the largest conserved savanna area in South Africa the Kruger National Park (KNP) plays a significant role in the conservation of savanna biodiversity, and reduction in its carrying capacity for grazer or browser species threatens the conservation effort.

A number of factors influence the abundance of woody vegetation on savannas. However, fire and herbivores are the primary determinants of the woody vegetation (Sankaran et al. 2005; Holdo et al. 2009; Accatino et al. 2010). Fire is a major disturbance to savanna woody vegetation through physical damage to woody plants. The effects of fire vary depending on the characteristics of the fire in terms of fire season (time of year), fire frequency and intensity. If sufficiently frequent in relation to the growth and regeneration rates of fire-intolerant woody species, fire prevents them from reaching high abundance (Skarpe 1991). Herbivores, through physical damage, have effects on variables like species composition, height, canopy size, stem diameter, and density (Levick and Rogers 2008; Wigley et al. 2014). Compared to other herbivores, mega-herbivores like elephants have been pointed out to have the largest impact on the woody vegetation (de Beer et al. 2006).

Rainfall and soil properties also influence savanna woody vegetation (Sankaran et al. 2008). Rainfall generally correlates positively with savanna woody cover up to a threshold of about 700 mm annual rainfall; and soil factors such as fertility and texture can act as limiting factors for some woody species (Sankaran et al. 2008). The woody vegetation and grass compete for water and soil nutrients (February and Higgins 2010; Rossatto et al. 2014), among other resources. Factors that can help tip the balance in favour of either grass or woody vegetation can have a significant influence on savanna woody vegetation. Skarpe (1991) suggests that in arid and semi-arid savannas competition for soil moisture is the main determinant of the woody component. In that situation the elimination of the grass as a competitor, e.g. through high grazing pressure, results in more water in both deeper and surface soil becoming available for woody growth (Skarpe 1991). Experiments by Kulmatiski and Beard (2013), on the other hand, showed that without changing the total amount of precipitation, small increases in precipitation intensity can push water deeper into the soil, increase savanna aboveground woody plant growth and decrease grass growth.

The effect of the determinants of woody vegetation structure and abundance varies depending on the savanna site. Thus, savannas can be either climate or disturbance dependent ecosystems depending on the environment in which they are located (De Michele et al. 2011). In the Kruger National Park, elephants and fire have been recognised to have significant effects on the woody vegetation (Trollope et al. 1998; Brits et al. 2002; Higgins et al. 2007). Owen-Smith et al. (2006) have argued that the effect of elephants on woody vegetation in the KNP has been exaggerated. Enslin et al. (2000) established that the effects of fire varied depending on the climate, herbivory, and soils of the location in the KNP. Shackelton and Scholes (2000) determined that increasing fire frequency significantly decreased woody basal area, biomass, density, height, mean stem circumference, and number of stems per plant in the KNP; while the proportion of regenerative stems increased with increasing fire frequency. Fires that occur in the late dry season (i.e. in spring) reduce woody vegetation cover the most (Smit et al. 2010). From plots in the KNP that had been exposed to long-term experimental burning Higgins et al. (2007) established that, although the woody vegetation appeared demographically resilient to fire, the relative dominance of small savanna trees was highly responsive to the fire regime.

In the long term, continued disturbance of the savanna woody vegetation due to one or more of the influencing factors could result in change in woody vegetation cover. Historical imagery serves as a data source to enable the detection of this change. Historical aerial photographs can supplement satellite imagery for this purpose, given that satellite imagery is only available since the early 1970s, and high spatial resolution satellite images only since the late 1980s. Historical aerial photographs have the advantage of high spatial resolution, while satellite images are advantageous over aerial photographs in that they enable synoptic coverage of large areas that would require multiple aerial photographs to cover. A number of studies analysing change in woody cover on savannas have used historical aerial photographs either as a photo only imagery time series (e.g. Hudak and Wessman 1998; Fensham and Fairfax 2003) or in combination with satellite images (e.g. Hudak and Wessman 2001). Appropriate algorithms to delineate the woody vegetation on the imagery are required, particularly when a linkage between historical aerial photographs and satellite images is being established.

Analyses of change in woody cover in the KNP using historical imagery have yielded mixed trends. Using manual photo interpretation Trollope et al. (1998) compared the density of large trees for the periods 1940 versus 1960 and 1960 versus 1986/89 on four of the major vegetation landscape units in the KNP. The results indicated that there were no significant changes in vegetation located on granitic soils between 1940 and 1960, whereas a moderate decline occurred in the areas with basaltic soils. Conversely, there was a dramatic decline in the density of large trees in all four major vegetation landscape units between 1960 and 1986/89, the decline attributed to a sharp increase in the elephant population as well as the introduction of burning programs (Trollope et al. 1998). Eckhardt et al. (2000) utilised quantitative analysis in a Geographic Information System (GIS) in assessing change in woody (tree and shrub) cover in a central strip of the KNP, using aerial photographs dated 1940, 1974 and 1998. The results showed a 12 % increase in woody cover on granite substrates but a 64 % decrease on basalt in the 58-year period.

This study assessed change in savanna woody cover at large study sites in the Kruger National Park, in a GIS framework that facilitated quantitative analysis. Unlike the previous similar studies, the present study used a longer time period (1940/42–2012) and relatively larger study sites.

## Methods

### Design: study sites

**Fig. 1:**
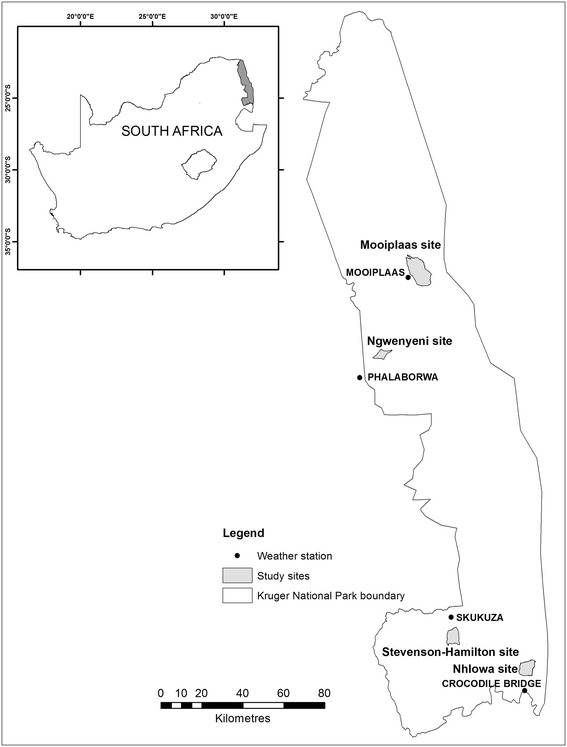
Location of the study sites in the Kruger National Park

The study sites were the four research supersites in the Kruger National Park (Ngwenyeni, Mooiplaas, Nhlowa, Stevenson-Hamilton; Fig. 1), that are described by Smit et al. (2013a). Northern parts of the KNP receive lower rainfall than the south (Gertenbach 1983; Viljoen 1995). The geology of the KNP broadly consists of basalts in the eastern half and granites in the west. The KNP research supersites are representative of these rainfall and geology characteristics (Smit et al. 2013a). The northern study sites (Ngwenyeni and Mooiplaas) receive lower rainfall than the southern sites (Nhlowa, Stevenson-Hamilton). The Nhlowa and Mooiplaas sites are on basalts, while the Ngwenyeni and Stevenson-Hamilton sites are on granites.

### Field quantification of woody cover

Field quantification of woody cover was undertaken in 2013 and 2014 at the four study sites, in order to derive data on woody cover for use in image interpretation. Sample plots measuring 100 m × 100 m (1 ha) were used during the field quantification. Rather than visual estimation of woody cover as has been employed by some studies, more reliable quantification was sought. Therefore, a method for estimating the total area covered by woody canopies in the sample plots was devised. The canopy diameter (*d*) for each woody individual (tree, shrub) in the plot was determined by spreading measuring tape either above (for shrubs) or below the canopy. Treating the canopy as circular, the *d* value then gave a radius, *r* (i.e. *r* = 1/2*d*), and the area covered by the canopy was then computed as: *Area* = *πr*^2^. The individual canopy area values were then summed to derive the total area under woody canopies, and the woody fraction of cover derived as a percentage of the 1 ha area of the plot. The coordinates of the corners and centre of the plot were then taken, using a Garmin eTrex hand-held Global Positioning System (GPS) that had accuracy of 3 m. These coordinates were then used in locating the sample plots on images of the respective study sites.

Woody cover in the KNP has been shown to vary depending on topographic position, with the valleys having more woody cover (Gertenbach 1983). The topographic location of the field sample plots was, therefore, purposefully varied in order to represent woody cover in valley, crest and mid-slope positions. Therefore, purposive sampling was employed in the field, with the goal of representing these topographic positions in the sample data. The overall layout of the sample plots at each of the study sites approximated linear transects. Logistics and the time-consuming nature of the field work limited the total number of sample plots and necessitated separate fieldwork excursions to the study sites during 2013 and 2014. The final total number of sample plots was 28:8 each at the Stevenson-Hamilton and Nhlowa sites, and 6 each at the Mooiplaas and Ngwenyeni sites.

### Historical imagery

The 1940/1942, 1968 and 1977 historical aerial photographs of the respective research supersites that are listed by Smit et al. (2013a) served as starting point in selecting historical imagery for use. These historical aerial photographs are all panchromatic, i.e. derived from photographic film that is sensitive in the broad spectral range 0.4–0.7 μm (blue, green, red) and depicts features in shades of grey. They were obtained from the National Geospatial Information (NGI) in Cape Town, South Africa.

For the period after 1977 imagery of the sites was selected from the catalogue of SPOT (***S****ytéme****P****our l’****O****bservation de la****T****erre*) images at the South African National Space Agency (SANSA). Aerial photographs generally have high spatial resolution. Therefore, SPOT images were judged to be more ideal for use in conjunction with the photographs than Landsat images that date back to the 1970s, based on the spatial resolution criterion. However, SPOT images are only available since the launch of the first SPOT satellite on 21 February 1986. Therefore, SPOT images of the late 1980s were selected, and then images from the late 1990s so as to approximate the decade inter-image date sequence of the 1968 and 1977 aerial photographs. However, SPOT images of the late 1980s (acquired from the SPOT 1 satellite), though listed on the SANSA catalogue, could not be supplied by SANSA due to technical difficulties. The final set of imagery was SPOT images acquired close to (or in the same season immediately after) the respective dates of the woody cover quantification fieldwork in 2013 and 2014 at the respective sites.

Panchromatic SPOT images (spectral sensitivity 0.51–0.73 μm) were ideal, because the historical aerial photographs were panchromatic. The aerial photographs had April, June and August dates in the respective years. As a consequence, the selection of SPOT images sought images that were as close as possible to the photo acquisition months, within the constraints of image availability on the SANSA catalogue.

The combined list of the imagery that was used is shown in Table 1. Not all the aerial photographs listed by Smit et al. (2013a) for the respective sites were selected for use in this study, because of the approximately 60 % end-lap and 28 % side-lap on aerial photographs. The overlap areas were rather problematic because of image parallax. Image parallax introduced an apparent change in relative positions of woody individuals due to change in the photographing station along the flight strips, resulting in some woody individuals being obscured on one of the overlapping photos. Therefore, stereopairs of photographs were avoided where possible, with synoptic and complete coverage of the study site as the overriding criteria. Excluding the overlap areas also helped avoid regions of the photographs with high relief displacement. Relief displacement resulted in features with height (e.g. tall trees) tending to lean away from the centre (principal point) of the photograph.

**Table 1:** List of imagery used

Study site (Fig. 1)	Aerial photographs (see Fig. 2)			SPOT images (see Fig. 2)				Fieldwork dates
	Dates	Scale	Photo numbers (codes)	Date (SPOT satellite)	K/J reference	Spectral sensitivity	Spatial resolution (m)	
Stevenson-Hamilton	1940	1:21,000–1:35,000	155-009-00454 155-009-00455	6 October 1998 (SPOT 2)	139/400	Panchromatic	10	
	18 June 1968	1:64,000	539-018-01243	5 April 2012 (SPOT 4)	139/400	Red	10	June, September 2013
	15 April 1977	1:30,000	788-002-00020 788-003-00112 788-003-00113					
Nhlowa	1940	Severe burn scars; photos not used.		12 June 1998 (SPOT 4)	140/400	Red	10	
	18 June 1968	1:64,000	539-020-01522					
	15 April 1977	1:30,000	788-006-00140 788-006-00142 788-007-00206 788-007-00207	9 June 2012 (SPOT 5)	141/401	Panchromatic	5	June, September 2013
Ngwenyeni	August 1942	1:30,000	165B-031-59497 165B-031-59498 165B-032-59478 165B-032-59479	15 July 1998 (SPOT 2)	138/398	Panchromatic	10	
	1968	1:64,000	539-004-00613					
	April 1977	1:30,000	779-023-02740 779-024-09461	5 April 2012 (SPOT 4)	138/398	Red	10	May 2014
Mooiplaas	August 1942	1:30,000	165B-016-60016 165B-016-60017 165B-017-59965 165B-017-59966 165B-017-59968 165B-018-59939 165B-018-59941	15 July 1998 (SPOT 2)	138/397	Panchromatic	10	
	1968	1:64,000	539-001-00546	7 April 2012 (SPOT 5)	138/397	Panchromatic	5	May 2014
	April 1977	1:30,000	779-002-05224 779-008-02899 779-008-02900					

**Fig. 2:**
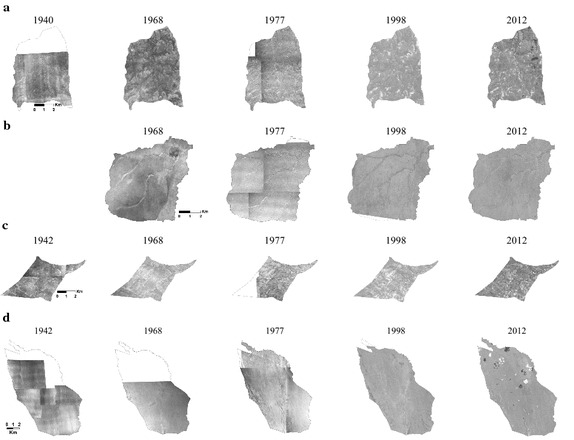
Coverage of unprocessed image subsets of the study sites shown in Fig. 1 on the different image dates in Table 1. **a** Stevenson-Hamilton site, **b** Nhlowa site, **c** Ngwenyeni site, **d** Mooiplaas site

For some of the study sites, the aerial photographs listed by Smit et al. (2013a) did not cover the entire research supersite. Nearly all the study sites had incomplete photographic coverage on at least one date. The portions of the study sites without photographic coverage (see Fig. 2) were subsequently omitted from the final combined analysis of woody cover change, because no photographs were available even at the NGI. For the Nhlowa site the 1940 photos depicted extensive burning of the site and were, therefore, not used in the analysis. At the 1:64,000 scale of the 1968 photographs the respective study sites could be covered by the respective single photos specified in Table 1. However, for the Mooiplaas site the 1968 photograph was apparently at the northern edge of photography job number 539. Therefore, only the southern half of the site was covered, as could be verified on a flight diagram obtained from the NGI.

### Image pre-processing

The aerial photographs were scanned at the NGI, using an Epson Expression 10,000 XL scanner with quite high resolution (1200 dots per inch). Since the scanning was performed at the NGI technical issues at this stage, such as the optimisation of tonal variation, could not directly be controlled. In some similar work (e.g. Hudak and Wessman 1998) the scanner resolution is altered depending on the photo scale.

The output pixel (grain) size on scanned aerial photographs can be computed using the formula in Eq. 1 (Hudak and Wessman 1998):

1 $${\text{Grain}}\;{\text{size}}\;({\text{m}}) = \frac{{{\text{Photo}}\;{\text{scale}}}}{{{\text{Scanner}}\;{\text{resolution}} \times 39.37\;{\text{inches}}/{\text{m}}}}.$$

Based on Eq. 1, the scanned photos all had pixel sizes of less than 2 m (0.44–1.35 m) at the respective scales listed in Table 1. After the delineation of woody cover on the respective photos these pixel sizes were later degraded to that of the lowest resolution SPOT images (i.e. 10 m). Retaining the high spatial resolution during mapping of the woody cover was important because some savanna woody cover crowns are less than 1 m in diameter.

The aerial photographs and SPOT images were imported into ERDAS Imagine 2014^®^ software, and georeferenced to the UTM projection using ground control points (GCPs) that were well spread in the image scenes. The GCPs mainly consisted of road and power line junctions. During georeferencing low rectification error (expressed as Root Mean Square (RMS) error) was aimed at. Due to the large number of aerial photograph and satellite image scenes, the individual RMS error values were too many to individually report but all were less than one pixel. On the older photographs the search for GCPs was rather challenging due to the relatively fewer roads and power lines that existed in the KNP then. This limited the number of GCPs to 7–9 on the old aerial photographs, in particular the 1942 photographs of the Mooiplaas site. For this reason the 1942 aerial photographs of the north-eastern sector of the Mooiplaas site could not be georeferenced and were, therefore, not used. After the georeferencing, shapefiles of the study sites were used in subsetting the images to yield the images depicting the study sites on the respective dates shown in Fig. 2. The 1940/42, 1968 and 1977 scenes in Fig. 2 are aerial photograph frames of the respective study sites prior to further processing and the creation of mosaics. The Mooiplaas site had scattered clouds in its northern half on the 2012 image (Fig. 2d).

Overlapping sections of the aerial photographs were carefully trimmed out in order to minimise image parallax, using image subsetting procedures within ERDAS. In order to optimise the contrast between the light and dark tones, contrast on each trimmed image was enhanced by spreading the digital number (DN) values on the full grey level scale range using a linear stretch. After separately mapping the woody cover on the respective aerial photographs from a given analysis year and study site, using image classification, the photographs were joined into thematic layer mosaics. All the images were carefully examined for presence of burn scars whose dark tone could have introduced error in mapping woody cover. Consequently the 1940 photographs of the Nhlowa site were excluded from the analysis due to extensive burn scars.

The SPOT images that were used were acquired by the different sensors (HRV, HRVIR, HRG) on board SPOT satellites 2, 4 and 5 (Table 1). There are sensor calibration differences among the SPOT sensors that can be minimised using inter-sensor calibration algorithms (Meygret 2005). In addition the HRVIR sensor (on board SPOT 4) does not have a true panchromatic band but a “Red” band in black and white, with spectral sensitivity as 0.61–0.68 μm compared to 0.51–0.73 μm on the HRV (SPOT 1, 2) and 0.48–0.71 μm on the HRG (SPOT 5) panchromatic bands. The mapping of woody cover on the images used photo texture (supplemented by interpretation of tone) and not direct comparison of DN values. Therefore, the error introduced by the sensor differences was judged as having little effect on the accuracy of mapping the woody cover.

### Mapping change in woody cover on the historical imagery

Photo texture has commonly been used in analyses of woody vegetation on aerial photographs and satellite images (e.g. Hudak and Wessman 1998, 2001; O’Connor and Crow 1999; Asner et al. 2003). In digital analyses of woody cover, image texture is employed as an enhancement method prior to mapping the woody cover through image classification. Woody vegetation has coarse texture, while grass has smooth texture.

Texture analysis was employed in delineating the woody cover in this study, using the texture analysis function in ERDAS. This function enhances texture by computing new DN values based on variance, skewness, kurtosis or Mean Euclidean Distance using DN values in an *n* × *n* moving window (where *n* is an odd number, i.e. 3, 5, etc.). The texture window should be smaller than the smallest feature being analysed, and this is optimised by high spatial resolution (Baraldi and Parmiggiani 1995; Hudak and Wessman 1998). Therefore, in this study the smallest window size 3 × 3 was judged to be optimal so as to detect small crown woody vegetation. After visually examining the texture images produced by each of the four operators in the texture analysis function the Mean Euclidean Distance operator was judged to be the most suitable option in comparison with variance, skewness, and kurtosis. Within the 3 × 3 window the Mean Euclidean Distance (MED) operator used the difference (‘distance’) in DN value between a given pixel and the central pixel, and replaced the central pixel DN with the average (mean) difference as in Eq. 2 (Irons and Petersen 1981):

2 $$MED = \frac{{\sum {\left[ {\sum\nolimits_{\lambda } {\left( {x_{c\lambda } - x_{ij\lambda } } \right)^{2} } } \right]^{{\frac{1}{2}}} } }}{n - 1}$$

where *x*_*ijλ*_ = DN value for spectral band *λ* and pixel (*i*,*j*) of image, *x*_*cλ*_ = DN value for spectral band *λ* of a window’s centre pixel, *n* = window number of pixels (=3).

Therefore, features with high reflectance (such as bare soil) had high MED values (due to high DN values) and low reflectors (such as woody vegetation) had low values (due to low DN values) on the resulting MED enhanced image. The contrasts were used in classifying woody vegetation on the resulting texture images.

On the resulting texture images, woody vegetation was then delineated by automated clustering using the *K*-means algorithm in ERDAS. As a way of reducing error the automated clustering was preferred to setting thresholds on the texture images, which has been employed in some studies (e.g. Hudak and Wessman 1998, 2001). Following clustering, the next step was to identify the woody cover cluster. Image tone at the respective locations on the original images was employed in interpreting the resulting texture images in order to identify the woody vegetation cluster. Three tones could be identified on the original images (Fig. 2): the bright tones of bare surfaces (dry soil, sand, and gravel), the light grey tones of senescent vegetation (mainly herbaceous grass) and the darker shades of grey depicting healthy vegetation. Since the image dates (Table 1) were all largely dry season dates, the healthy vegetation was interpreted as woody vegetation. Pixels with woody vegetation had low Mean Euclidean Distance values on the texture images. On the other hand dry sand on river beds as well as senescent herbaceous vegetation had higher Mean Euclidean Distance values. *K*-means clustering requires prior knowledge of the number (*K*) of clusters, unlike the ISODATA algorithm (Selim and Ismail 1984; Jain 2010; Yildirim 2014). Therefore, the three image tone based classes (dry, bare surface; senescent herbaceous vegetation; woody vegetation) were specified for clustering.

Woody vegetation had dark tones due to chlorophyll’s high absorption in the blue and red spectral ranges. Dry features like sand on the other hand reflect highly in blue, green and red, resulting in their brighter tones on either the panchromatic or the grey scale red band images. Unfortunately, shadow and burn scars introduced error in that burn scars and dry features that were in shadowed locations also had dark tones. These features tended to have Mean Euclidean Distance values that were similar to those of woody vegetation on the texture images. The dark edges of the photographs introduced further error and were, therefore, not included in the final woody vegetation thematic layers.

The classified images needed assessment of classification accuracy. Ideally assessment of classification accuracy is performed by generating random coordinates and, using a contingency table (an error matrix), comparing the classifications of the randomly sampled sites with their field (reference) classes (Foody 2002). For the newest (SPOT) images classification accuracy was assessed using, as reference data, georeferenced digital 0.5 m resolution colour aerial photographs of the study sites that were acquired in 2010. On the 2012 classifications the respective overall classification accuracies for the Nhlowa, Mooiplaas, Ngwenyeni and Stevenson-Hamilton sites were 89, 91.5, 93.8 and 95 % (Table 2a), which indicated that the texture enhancement based classification scheme generally had high accuracy. For the older images assessing classification accuracy was rather problematic due to the lack of reference data. However, an indication of the accuracy of classification was obtained by generating a stratified random sample of 50 points per respective classified image, and then the presence of woody vegetation at each of the assessment points was confirmed or refuted by an independent analyst. The indicative classification accuracy values are summarised in Table 2b. In general the classifications of the 1968 photographs were least accurate, due to the low photo scale of 1:68 000. The 1977 aerial photographs were of better print quality which made identification and classification of woody vegetation easier, resulting in comparatively higher classification accuracy (Table 2b). Despite their higher spatial resolution the aerial photographs generally had lower classification accuracy due to print quality.

**Table 2:** Indicative classification accuracy of the multiple date images of the study sites

	Overall classification accuracy (%), per study site			
	Stevenson-Hamilton	Ngwenyeni	Nhlowa	Mooiplaas
(a) SPOT images				
SPOT, 2012	95.0	93.8	89.0	91.5
SPOT, 1998	90.3	89.1	92.4	88.9
(b) Aerial photographs				
1940/1942	74.9	80.2	^a^	86.8
1968	70.2	75.7	79.7	74.3
1977	84.6	87.3	89.1	88.7

^a^Photos not used due to severe burn scars

Change in woody vegetation cover was then detected by GIS overlay analysis in ERDAS, using Boolean analysis. Boolean analysis employs arithmetic cell to cell comparisons of co-registered raster data sets. In order to accomplish this, the spatial resolution of all the extracted woody cover thematic layers was reduced to 10 m, the pixel size of the lowest resolution images in the data set. Numeric codes were then assigned to the woody cover thematic layers per date for the respective study sites. The codes 3, 5, 7, 11, 17 were used for 1940/42, 1968, 1977, 1998, 2012 woody cover, respectively. These codes gave unique resulting codes using Boolean addition for the GIS overlay analysis, compared to the coding sequence of 1, 2, 3, 4, 5. For example, 8 on the thematic layer resulting from the Boolean addition overlay meant woody cover in 1940/42 (coded as 3) and 1968 (coded as 5) only and none thereafter, 10 meant woody cover in 1940/42 (coded as 3) and 1977 (coded as 7) only, etc. The overlay analysis used intersects of the respective study areas, i.e. only the sections of the respective study sites that had image coverage on all the respective image dates in Fig. 2. Based on the area covered by a 10 m pixel the change in woody cover between dates could be quantified in ERDAS, and then mapped using the Geographic Information System software ArcMap 10.2.

## Results

### Field data on woody cover

Field data obtained in 2013 and 2014 confirmed the difference in woody cover on granite and basalt substrates in the KNP that is indicated by Gertenbach (1983). The granite study sites had higher woody cover than the basalt sites and their woody cover was characterised by high variance (Table 3a). The difference in woody cover was statistically significant between the Ngwenyeni (northern granites) and Mooiplaas (northern basalts) sites (*t* = 3.68, *P* = 0.008), and between the Stevenson-Hamilton (southern granites) and Nhlowa (southern basalts) sites (*t* = 2.54, *P* = 0.029; Table 3b).

**Table 3:** Study site woody cover statistics from field data in 2013 and 2014

Study site	Mean woody cover (%) per hectare		
	Overall	By topographic position	
(a) Mean woody cover and variance (*s* ^*2*^)			
Stevenson-Hamilton (southern granites)	25.8 (*s* ^2^ = 287.7)	Crest	20.5
		Mid-slope	17.8
		Valley	39.2
Ngwenyeni (northern granites)	34.2 (*s* ^2^ = 191.7)	Crest	26.7
		Mid-slope	28.4
		Valley	47.5
Nhlowa (southern basalts)	9.3 (*s* ^2^ = 53.4)	Crest	11.0
		Mid-slope	2.1
		Valley	14.7
Mooiplaas (northern basalts)	10.9 (*s* ^2^ = 49.3)	Crest	9.7
		Mid-slope	8.7
		Valley	14.3

	Stevenson-Hamilton	Ngwenyeni	Nhlowa
(b) Statistical significance of differences in site overall mean woody cover			
Ngwenyeni	*t* = 1.78, *P* = 0.379 (*NS*)		
Nhlowa	*t* = 2.54, *P* = 0.029*	*t* = 3.88, *P* = 0.006**	
Mooiplaas	*t* = 2.37, *P* = 0.039*	*t* = 3.68, *P* = 0.008**	*t* = 0.21, *P* = 0.836 (*NS*)

*NS* = not significant at 5 %; * significant at 5 %; ** significant at 1 %

For similar geological substrates, there was higher woody cover in the north compared to the south (Table 3a). The highest mean woody cover was at the northern granites (Ngwenyeni) site (mean = 34.2 %), followed by the southern granites (Stevenson-Hamilton) site (mean = 25.8 %), then the northern basalts (Mooiplaas) site (mean = 10.9 %), and the southern basalts (Nhlowa) site (mean = 9.3 %). In terms of topographic position, the valleys generally had the highest woody vegetation cover. The Ngwenyeni site’s valleys generally had higher woody cover (mean ≈ 48 %) than the Stevenson-Hamilton site (mean ≈ 39 %). Mid-slope positions had lower woody cover that crest positions on basalt (Table 3a).

The differences in woody cover among the four study sites are noticeable on the image subsets in Fig. 2. The less wooded Nhlowa and Mooiplaas sites have smooth-textured images (Fig. 2b, d, respectively), whereas the more wooded Stevenson-Hamilton and Ngwenyeni sites have rather coarse texture (Fig. 2a, c, respectively). Woody vegetation is also distinguishable on the images in Fig. 2 based on its dark tones, especially in the more wooded valleys. The image sensor spectral differences, for example between the SPOT 4 HRVIR ‘red’ grey scale images and the SPOT 2 and SPOT 5 panchromatic images, do not appear to affect this visual distinctness of the woody vegetation. The main discernible difference between the SPOT 4 HRVIR grey scale images on the one hand and the SPOT 2 and SPOT 5 panchromatic images is that on the SPOT 4 images woody vegetation is darker in tone (the 2012 vs. the 1998 images in Fig. 2a–c), due to the strong absorption of red energy by chlorophyll.

### Change in woody cover

**Fig. 3:**
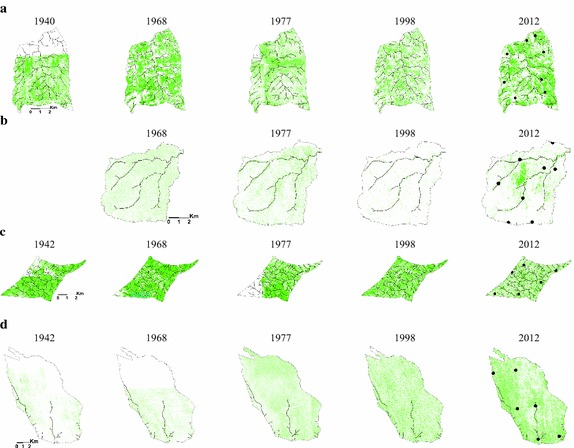
Extracted woody cover on the images of the study sites in Fig. 2, with streams (*lines*) and field sample sites (*dots*) superimposed. **a** Stevenson-Hamilton site, **b** Nhlowa site, **c** Ngwenyeni site, **d** Mooiplaas site

There were inter-date shifts in location and amount of woody cover, including apparent cycles (losses and gains) in the woody cover (Table 4; Fig. 3). In the long-term the results indicated different trends in woody cover between the two study sites in the north (Ngwenyeni, Mooiplaas) on the one hand and those in the south (Stevenson-Hamilton, Nhlowa). Indications from the image analysis were that, for the analysis period, the southern sites lost woody cover while the northern sites gained. This can be discerned visually on Fig. 3, where the woody cover per image data is mapped at the respective original pixel sizes. High intensity of tone (colour) on the thematic layers in Fig. 3 indicates a high density of pixels that had woody cover as detected by the mapping procedures, and vice versa. The woody cover extraction procedures correctly depicted the basalt sites (Nhlowa and Mooiplaas; Fig. 3b, d) as less wooded than the granite sites (Stevenson-Hamilton and Ngwenyeni; Fig. 3a, c), due to the inherent differences in woody cover that exist between these geological substrates (Gertenbach 1983; Table 3). At the small scales of Fig. 3 the intensity of tone erroneously gives the perception of thick woody cover in places, but at large scale the scattered nature of the cover was discernible.

**Table 4:** Area of cover of image classification classes in the sections of the study sites that were common on the image dates (as in Fig. 4)

Study site and imagery	Class and area of cover (ha), at original image pixel size		
	Woody vegetation	Senescent vegetation (grass)	Dry, bare surface
(a) Stevenson-Hamilton site			
1940 aerial photographs	1859.61	958.79	231.13
1968 aerial photographs	1615.89	1216.41	217.23
1977 aerial photographs	1400.53	1470.07	178.93
*1940*–*1977 change:* −*25.7* *%*			
1998 SPOT image	1353.69	1467.40	228.44
2012 SPOT image	1371.73	1553.84	123.96
*1998*–*2012 change:* +*1.3* *%*			
(b) Nhlowa site			
1940 aerial photographs^a^			
1968 aerial photographs	635.84	2478.99	1201.30
1977 aerial photographs	625.86	3233.47	456.80
*1968*–*1977 change:* −*1.6* *%*			
1998 SPOT image	528.91	3673.27	113.95
2012 SPOT image	397.69	3192.86	725.58
*1998*–*2012 change:* −*24.8* *%*			
(c) Ngwenyeni site			
1942 aerial photographs	489.52	1059.57	92.24
1968 aerial photographs	546.20	915.78	179.35
1977 aerial photographs	512.87	1073.62	54.84
*1942*–*1977 change:* +*4.8* *%*			
1998 SPOT image	744.32	799.71	97.30
2012 SPOT image	687.39	801.29	152.65
*1998*–*2012 change:* −*7.6* *%*			
(d) Mooiplaas site			
1942 aerial photographs	831.88	5737.62	237.39
1968 aerial photographs	1298.53	5399.63	108.73
1977 aerial photographs	1821.32	4655.71	329.86
*1942*–*1977 change:* +*119.0* *%*			
1998 SPOT image	1956.24	4323.47	527.18
2012 SPOT image	2640.69	3709.99	456.21
*1998*–*2012 change:* +*35.0* *%*			

^a^Photos not used due to severe burn scars

Scattered cloud affected the amount of woody cover that was mapped on the 2012 (see Figs. 2d, 3d), and a faint burn scar in the central sector of the Nhlowa site on the 2012 image (Fig. 2b) introduced the error of apparently high woody cover (Fig. 3b). Despite these minor errors in mapping the woody cover, general trends in long-term woody cover are noticeable. The extracted woody cover layers for the Mooiplaas and Ngwenyeni sites on the respective different dates showed a tone intensification trend from the oldest date to the newest, which indicated gradual gains in woody cover. The opposite was the case with the Nhlowa and Stevenson-Hamilton sites, indicating gradual losses in woody cover.

**Fig. 4:**
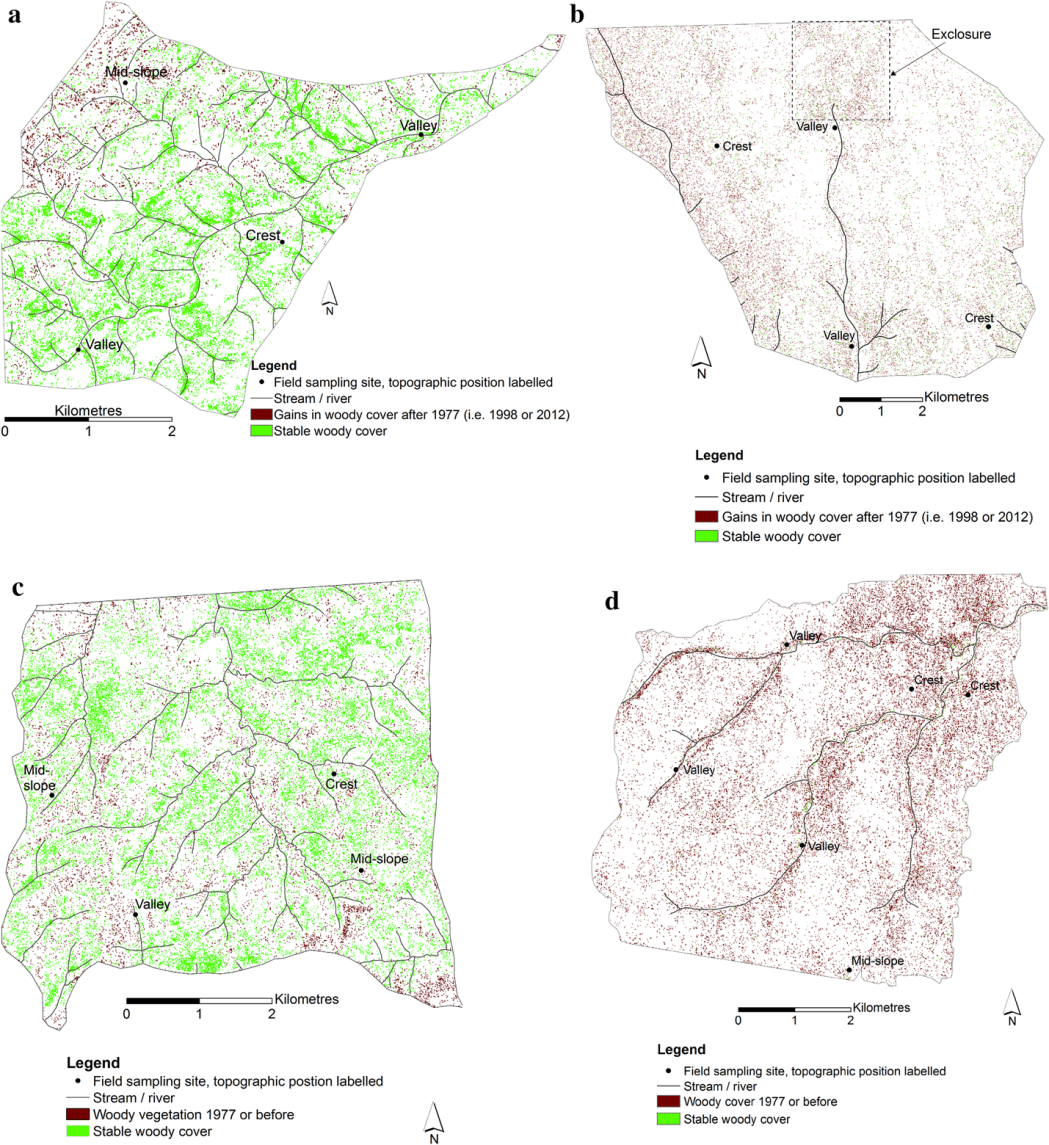
Illustration of change in woody cover that resulted from intersect GIS overlay analysis. **a**, **b** The study sites with long-term gains, and **c**, **d** the sites with long-term losses in woody cover. All sites had a core of stable woody cover (in *green*), as well as gained or lost woody cover (in *brown*). The overlay analysis used locations common to all dates on the respective extracted woody cover thematic layers of the study sites in Fig. 3. The woody cover is mapped here at the 10 m pixel size, which was the pixel size of the lowest resolution SPOT images. **a** Ngwenyeni site, **b** Mooiplaas site, **c** Stevenson-Hamilton site, **d** Nhlowa site

Nearly all image classification comes with elements of errors of commission and omission. Therefore, the amount of change in woody cover (positive or negative) may only be taken as indicative of the direction of change, with the actual values being subject to classification error. In Fig. 4 the change in woody cover that resulted from the GIS overlay analysis involving the imagery intersect locations for the respective study sites is mapped, at the pixel size of 10 m. Each of the study sites had a core of stable woody cover that was present on each of the imaging dates as well as locations with changed cover, as indicated on Fig. 4. Due to the variety of inter-date changes that resulted from the GIS overlay analysis, only the more sustained indicative changes in woody cover are indicated on Fig. 4 and summarised in Table 5. As a result of over-estimation introduced by pixel aggregation to the 10 m size, the actual long-term change % values are slightly different between Tables 4 and 5, although the respective trends are the same. Within the same study site, site-specific factors such as destruction by fire prior to an earlier date could help account for the apparent gains in woody cover between dates in Table 4 (e.g. between 1977 and 1998 at the Nhlowa site).

**Table 5:** Summary of overall change in woody cover at the study sites in relation to possible causes

Site	Location (geology)	Change in woody cover in section of study site in Fig. 4	Elephant density, individuals/km^2^ (Smit et al. 2013a)	Mean fire return interval, years (Smit et al. 2013b)	Average rainfall, mm/annum (Smit et al. 2013a)	Correlation between woody cover and cumulative rainfall in 10-year moving average analysis period in Fig. 5
Nhlowa	Southern basalts	−40 % (period: 1968–2012)	0.17	4.05	610	*r* = 0.533, *P* > 0.05, NS*
Mooiplaas	Northern basalts	>100 % (period: 1942–2012)	0.07	4.57	480	–
Stevenson-Hamilton	Southern granites	−29 % (period: 1940–2012)	0.09	5.80	560	*r* = 0.478, *P* > 0.05, NS*
Ngwenyeni	Northern granites	+39 % (period: 1942–2012)	0.22	9.39	490	*r* = −0.674, *P* > 0.05, NS*

* *NS* = Not significant at 5 %

– Not analysed due to short rainfall record

At the Ngwenyeni site, the stable woody cover was mainly in valleys and their vicinity (Fig. 4a). Away from the valleys, woody cover gains occurred between 1998 and 2012 in mid-slope and crest topographic positions, particularly in the site’s northern sections. In these sections of the site a large number of recruiting woody shrubs, mainly *Combretum* species, were observed during the fieldwork in May 2014. From the image analysis the indicative gain in woody cover between 1942 and 2012 was 39 % at this northern granites site.

The imagery intersect section of the Mooiplaas site (Fig. 4b) had a more than 100 % gain in woody cover between 1942 and 2012. This northern basalts site includes an exclosure (the Capricorn Rare Antelope Exclosure), whose high woody cover was successfully delineated by the woody cover mapping procedures. Established in 2002, this 500 ha exclosure restricts the entry of large herbivores and fire, and has contributed to the growth in woody cover in the site between 1998 and 2012. The southern basalts (Nhlowa) site on the other hand showed a loss of about 40 % in woody cover between 1968 and 2012. The Nhlowa site had very little stable woody cover, including in valley topographic positions (Fig. 4d). The locations with losses in woody cover after 1977 were scattered almost evenly across the site.

The Stevenson-Hamilton site’s imagery intersect section had stable woody cover in nearly all topographic positions, but underwent a general trend of loss in woody cover between 1940 and 2012. The loss in woody cover after 1977 at this southern granites site was nearly uniformly scattered in all sections of the site (Fig. 4c). Between 1942 and 2012 the loss in woody cover was about 29 % for the imagery intersect section of the site.

## Discussion

Possible causes of the change in woody cover at the four study sites can be interpreted by assessing the context-specific determinants of savanna woody cover. Since study sites on similar soils (basalt and granite, respectively) had different directions of change in woody cover (Table 5), soil type appears not to have contributed to the change.

**Fig. 5:**
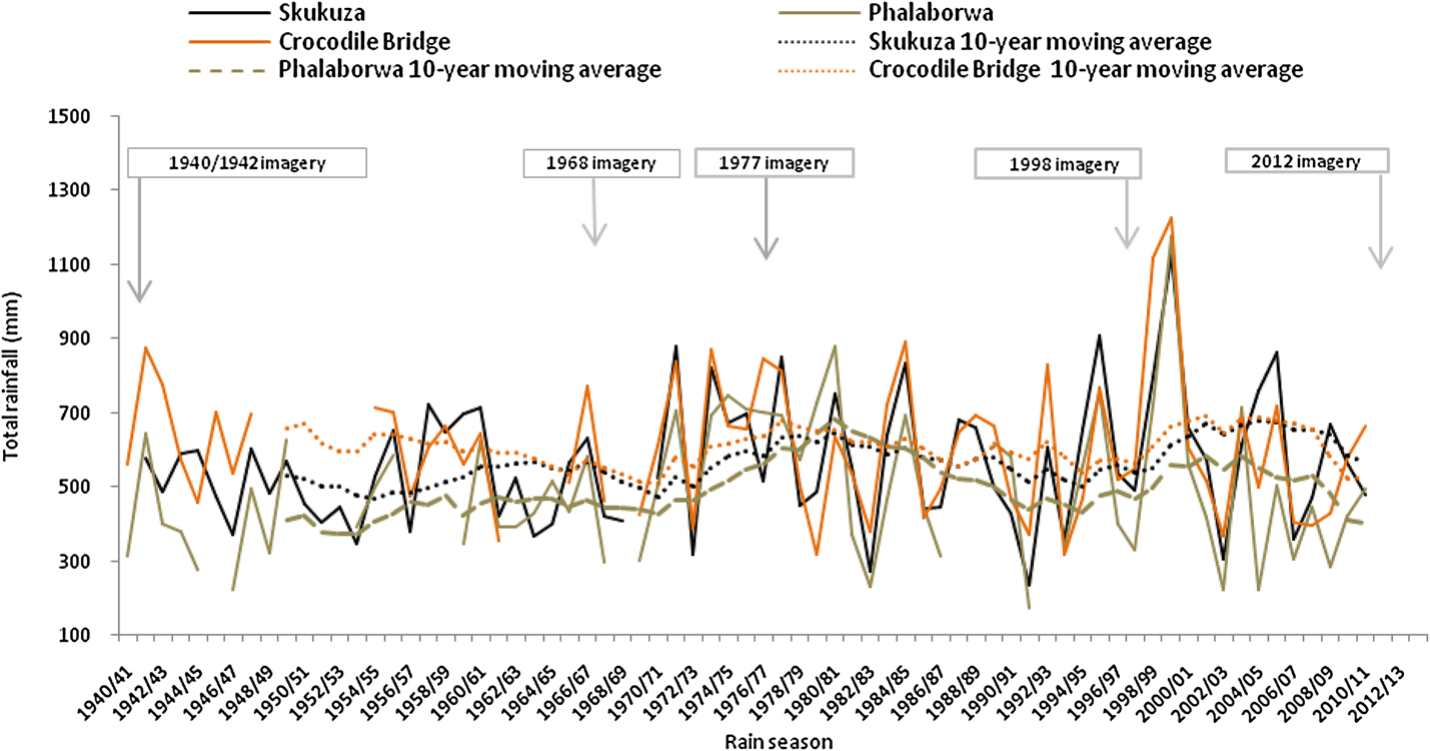
Imagery dates in relation to seasonal rainfall totals trends at the weather stations close to the study sites in (Crocodile Bridge, Phalaborwa, Skukuza; Fig. 1). Breaks in total rainfall graphs due to missing or incomplete records. *Data*: Smit et al. (2013a)

Ten-year moving average trend analysis of 1940-2011 total annual rainfall recorded at weather stations close to the study sites showed that the seasonal rainfall had phases of high followed by low rainfall (Fig. 5). The Mooiplaas station had records only from the 1974/75 to the 2010/11 rain seasons. Therefore, for this station 10-year moving average trend analysis was not performed. In addition to the wet and dry phases the moving average trend analysis showed that in the long-term the rainfall increased slightly since 1940 (Fig. 5), which should have resulted in an increase in woody cover at all of the study sites. Viljoen (1995) suggests that the woody vegetation in the northern sector of the KNP is adapted to drought; which suggests that low rainfall alone is unlikely to cause reduction in woody cover. Table 5 shows that there were non-significant (*P* > 0.05) Pearson’s correlation coefficient (*r*) values between the woody cover and the pre-image 10-year rainfall (i.e. rainfall for the 10-year period leading up to the woody cover determined on the respective images). For the Ngwenyeni site the relationship (*r*) between the rainfall and woody cover was negative; the woody cover increased despite the reducing rainfall. The southern sites had losses in woody vegetation despite their higher annual rainfall (Table 5). Site differences in rainfall and the long-term fluctuations in the rainfall, therefore, do not appear to have caused the gain in woody cover in the northern study sites.

Studies in African savannas have shown that the combination of elephant and fire damage to woody vegetation is pivotal in influencing the abundance of the woody vegetation. Examples are studies in Hluhluwe iMfolozi Park in South Africa (Staver et al. 2009), western Zimbabwe (Holdo 2007), the Serengeti in Tanzania (Holdo et al. 2009), northern Mozambique (Ribeiro et al. 2008), a park in southern Zimbabwe bordering the KNP (Gandiwa et al. 2011), as well as the KNP (Trollope et al. 1998). Differences in elephant densities and fire frequency can, therefore, be suggested as the main causes of the changes in woody cover that were determined by this study. The respective elephant densities (based on 1987–1993 data) for the Nhlowa, Stevenson-Hamilton, Mooiplaas and Ngwenyeni sites were 0.17, 0.09, 0.07, and 0.22 individuals/km^2^ (Smit et al. 2013a; Table 5). Therefore, the Nhlowa site had high elephant density, which partly explains why it had the higher loss in woody cover of 40 % compared to 29 % for the Stevenson-Hamilton site. The Ngwenyeni site had higher elephant density than the Nhlowa site but it did not undergo loss in woody cover in the analysis period, indicating that elephant density was not the sole driver of the change in woody cover.

**Fig. 6:**
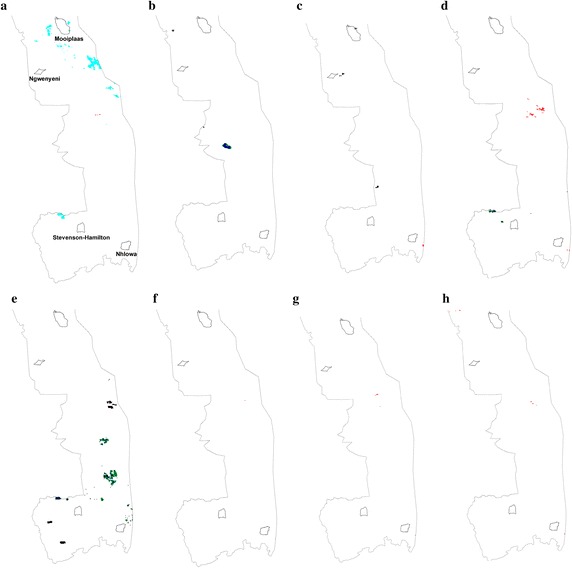
MODIS burned area monthly imagery at 500 m resolution (MCD45A1 data), in November of 2006–2013, showing fires (*red shades*) and possible fire-related features (aerosols/smoke: *blue*, *cyan*, *green colours*) in the section of the Kruger National Park where the study sites (named in (**a**)) are located. **a** 2006, **b** 2007, **c** 2008, **d** 2009, **e** 2010, **f** 2011, **g** 2012, **h** 2013. *Data*: USGS (2016)

The mean fire return intervals (based on 1941–2006 data) for the study sites as indicated by Smit et al. (2013b) were 4.05, 4.57, 5.80, and 9.39 years for the Nhlowa, Mooiplaas, Stevenson-Hamilton and Ngwenyeni sites, respectively (Table 5). Long-term fire frequency data by van Wilgen et al. (2000) for the period 1941–1996 indicate fire return periods of 4–5, 5–6, 6–7 and 7–9 years for the sections of KNP containing the Nhlowa, Mooiplaas, Stevenson-Hamilton and Ngwenyeni study sites, respectively. MODIS (MODerate resolution Imaging Spectroradiometer) burned area monthly images at 500 m resolution (MCD45A1 data) were used in this study to supplement the 1941–2006 period of fire frequency analysis in Smit et al. (2013b). MODIS burned area images for the date of 1 November each year in the period 2006–2013 were downloaded from EarthExplorer (USGS 2016), and then the KNP extracted from the scenes (no data before 2006 were available). November is at the culmination of the fire season in the KNP, just before the rains, and such late fires are the most destructive to woody vegetation (van Wilgen et al. 2000; Smit et al. 2010). The MCD45A1 image is based on 3 months of atmospherically- and geometrically-corrected, cloud-screened daily reflectance data (USGS 2016). Therefore, the MODIS fire images that were analysed spanned the period August, September and October. From the MODIS data no late fire occurred in the study sites themselves in the analysed period (Fig. 6), but there were fires in close proximity of the Nhlowa site in 2008–2013 (Fig. 6c–h), confirming its susceptibility to fire. There was also a fire event at the Mooiplaas site in 2008 (Fig. 6c).

Therefore, the Nhlowa site had the most frequent fires in addition to its high elephant density of 0.17 individuals/km^2^, which explains its loss of woody cover. The near-even spread of the woody cover losses at the Nhlowa site is consistent with elephant herbivory and destruction by fire. At the opposite end the Ngwenyeni site had the longest mean fire return interval (9.39 years), which explains its gain in woody cover. The gain in woody cover at the Mooiplaas site since 1942, despite relatively high fire frequency, can be attributed to the site’s low elephant density of 0.07 elephants/km^2^. Like the Ngwenyeni, the Stevenson-Hamilton site had low fire frequency (5.80 year mean fire return interval) and would, therefore, also be expected to have gained woody cover in the analysis period. The loss of woody cover at this site, therefore, appears to have resulted from destruction of woody plants by elephants. The scattered nature of the sites with woody cover losses since 1977 throughout the site (Fig. 4c), irrespective of topographic position, is consistent with elephant herbivory. The destruction of woody plants by the elephants was evident during the field work.

Such changes in woody cover threaten long-term shifts in relative distributions and abundance of trees and grass in savannas in general. Historical data and models have shown evidence of change in woody cover in savanna locations other than the KNP. For example, in the Hluhluwe iMfolozi Park thicket, forest, and densely wooded savanna now occur on sites that were previously grassland or open savanna (Gillson 2015). For the Serengeti, 100-year predictions by a model suggested stability in total woody cover at contemporary elephant densities of 0.15/km^2^ in the absence of fire, but that the mature tree population would decline regardless of the fire regime (Holdo et al. 2009).

For park management the results from this study point to the need for location context-specific management of fire and elephant concentrations. The changes in woody cover are likely to have effects on the grazer and browser carrying capacities of the savannas in the Kruger National Park. Gillson (2015) recommends the use of fire and grazers as conservation management intervention tools that can help maintain the balance between woody vegetation and grass on savannas. The Kruger National Park has a location context fire management policy that includes the reduction of woody encroachment and maintenance of grazing grass among its ecological management objectives (van Wilgen et al. 2014). As it was in 2013, however, the policy did not seem to have specific fire-related ecological management objectives for the riparian zone-adjacent areas in which the Ngwenyeni and Stevenson-Hamilton sites are located. Improved understanding of the effects of fire and its synergy with other savanna ecosystem drivers remained a challenge (van Wilgen et al. 2014).

Like the case with previous studies of change in woody cover using historical imagery of sections of the KNP (Eckhardt et al. 2000; Trollope et al. 1998), this study has yielded mixed trends. This suggests that long-term change in woody cover patterns in the KNP is location context-specific. Therefore, studying the change in woody cover using high spatial resolution imagery of the entire park is advisable in order to determine if location context does not influence the woody cover change trajectories. Using historical aerial photographs in a GIS environment such an undertaking is technically quite challenging due to a number of factors, including technical problems arising from scanning, the lack of synoptic coverage of large areas, and limitations in image processing algorithms that can function on the panchromatic photographs. Sub-pixel classification of woody cover, for example, was not feasible on the panchromatic photographs. Another technical problem is the vignetting (image fall-off) problem that typically occurs towards the margins of aerial photographs and is often amplified after scanning (Asner et al. 2003). In this study the dark patches on the edges of the photographs (see Fig. 2) were excluded from the eventual quantification of woody cover, which caused some of the blanks in the mapped woody cover in Fig. 3.

Using high spatial resolution satellite images, therefore, appears considerably advantageous. However the panchromatic aerial photographs remain a vital imagery record of the woody cover before the era of satellite imagery. In comparison with the aerial photographs, some of the satellite images that were used in this study had the lower spatial resolution of 10 m, which limited the detection of woody cover to crown sizes or woody crown clumps of widths greater than 10 m. It would have been advantageous to use the higher spatial resolution SPOT 5 images instead. However, SPOT 5 images are only available for dates after 2002. These differences in spatial resolution in the historical imagery necessitate mapping the woody cover at the high spatial resolution of the aerial photographs and then degrading the dataset to the resolution of the satellite images (e.g. Hudak and Wessman 1998). Automated mapping of woody cover on the photographs (which was employed in this study) has advantages over the commonly employed manual interpretation (Hudak and Wessman 2001).

## Conclusions

This study examined long-term (1940–2012) change in woody cover on the savannas of the Kruger National Park using study sites in northern and southern parts of the park. A combination of panchromatic aerial photographs and black and white SPOT images was used for the analysis. The results showed gains in woody cover at the northern granites and northern basalts sites, but losses at the southern granites and southern basalts sites. The combination of fire frequency and elephant densities accounts for most of the change in woody cover. This relative significance of fire and elephant damage in causing change in woody cover in the Kruger National Park, compared to other factors like rainfall, is in accordance with established theory of the pivotal influence of the two agents of change in savanna woody cover.

## Abbreviations

DN: digital number
GCP: ground control point
GIS: Geographic Information System
GPS: Global Positioning System
HRG: high resolution geometric
HRV: high resolution visible
HRVIR: high resolution visible infra red
ISODATA: iterative self-organising data analysis
KNP: Kruger National Park
MODIS: MODerate resolution Imaging Spectroradiometer
NGI: National Geospatial Information
RMS: root mean square
SANSA: South African National Space Agency
SPOT: *Sytéme**Pour l’**Observation de la**Terre*
UTM: universal transverse mercator
